# Current Landscape of IFN-λ: Induction, Inhibition, and Potential Clinical Applications to Treat Respiratory Viral Infections

**DOI:** 10.4049/immunohorizons.2200010

**Published:** 2023-04-18

**Authors:** Iván Martínez-Espinoza, Antonieta Guerrero-Plata

**Affiliations:** Department of Pathobiological Sciences, School of Veterinary Medicine, Louisiana State University, Baton Rouge, LA; Department of Pathobiological Sciences, School of Veterinary Medicine, Louisiana State University, Baton Rouge, LA

## Abstract

IFN-λ or type III IFN is an important mediator of antiviral response. Several respiratory viruses induce the production of IFN-λ during their course of infection. However, they have also developed intricate mechanisms to inhibit its expression and activity. Despite a considerable amount of research on the regulatory mechanisms of respiratory viruses on the IFN-λ response, little is still known about the effect of this cytokine on immune cells and the antiviral effects of all IFN-λ isoforms, and a better understanding of the detrimental effects of IFN-λ treatment is required. Here we highlight the relevance of IFN-λ as an antiviral cytokine in the respiratory tract. Data from studies in vitro, ex vivo, experimental animal models, and ongoing clinical trials emphasize the therapeutic opportunity that IFN-λ represents to treat and prevent different types of respiratory viral infections.

## Introduction

IFN-λ or type III IFN is a family of IL-10-related cytokines that evolutionarily are distantly related to type I IFNs (1). There are four known isoforms of IFN-λ: IFN-λ1, also known as IL-29; IFN-λ2 or IL-28A; IFN-λ3 or IL-28B (2); and IFN-λ4, which has been inactivated in many individuals by a frameshift mutation (3). Although IFN-λ4 exhibits antiviral activity similar to that of IFN-λ3 (4, 5), it is still debated whether IFN-λ4 induces cell death and decreases cell proliferation in tissues other than human hepatic cells (6) or if it causes no harm to epithelial cells (4). The induction of IFN-λ genes is regulated by specific pattern recognition receptors and signaling pathways that can activate the IFN-λ promoters (1, 2, 7). IFN-λ1 and IFN-λ2/3 promoters possess binding sites for both IFN regulatory factors (IRFs), and NF-κB transcription factors. As IFN-λ is expressed and released, it binds to its receptor in an autocrine or paracrine fashion (8), recognizing the IL-28RA subunit with high affinity and recruiting the IL-10RB subunit. Dimerization of the receptor leads to activation of the JAK-STAT pathway to induce different IFN-stimulated genes (ISGs) and the antiviral state in uninfected cells. All isoforms of IFN-λ have been shown to induce the expression of ISGs necessary for controlling viral infections (9–11). Furthermore, when identified as ISGs, IFN-λ1–3 can be induced strongly by stimulation from type I and type III IFNs (12), leading to the amplification of the IFN-λ response.

The antiviral effect of IFN-λ starts by binding to the receptor, which is composed of two subunits: the IFNLR1 or IL-28Rα-chain and the IL-10R2β-chain, which is shared with other cytokines in the IL-10 superfamily. IFNLR1 seems to be expressed in a tissue-dependent manner mainly in the stomach, intestine, and lungs and poorly in the CNS (13). Similar to type I IFN, IFN-λ can be rapidly induced after viral infections (14). However, unlike type I IFNs, which can induce a potent antiviral state in a wide variety of cells (i.e., epithelial cells, macrophages, endothelial cells, and lymphocytes, among others), IFN-λ antiviral protection is limited to a certain group of cells, such as epithelial cells, dendritic cells (DCs), neutrophils, and B cells, which have the highest expression of IFNLR1 (15–18). Thus, IFN-λ has been shown to contribute significantly to preventing viral invasion through mucosal surfaces (13). Additionally, experimental evidence has demonstrated that IFN-λ is highly expressed in epithelial cells with an elevated capacity to restrict the viral replication of different viruses (19) and modulate the immune response (16). Despite the importance of this antiviral cytokine, its therapeutic applications are still undeveloped. In this review, we summarize the progress toward understanding the mechanisms of activation and inhibition of IFN-λ during respiratory viral infections and the recent advances in its use in clinical settings.

### Induction of IFN-λ by respiratory viruses

In infected patients, the wild type and Omicron strain of severe acute respiratory syndrome coronavirus 2 (SARS-CoV-2) induces the expression of IFN-λ in the respiratory (20–22) and intestinal epithelia (23), which is important for controlling the infection (24, 25). However, the levels of IFN-λ2 in severe cases of coronavirus disease 2019 (COVID-19) have been reported to be lower than in the mild disease group (26), suggesting an important role of IFN-λ in the outcome of the disease and that the virus possesses mechanisms to inhibit IFN-λ expression. This would not be surprising, knowing the capacity of SARS-CoV-2 to inhibit the IFN-λ response, as detailed further below. Other respiratory viruses also induce an IFN-λ response. Evaluation of nasopharyngeal washes collected from infected children identified the expression of IFN-λ1, IFN-λ2, and IFN-λ3 by human respiratory syncytial virus (RSV) and rhinovirus (RV) (27). However, the role of IFN-λ expression in individuals infected with these respiratory viruses warrants further investigation into the mechanisms mediating the complex interplay between its expression and disease severity.

After viral recognition, most cells are able to produce IFNs rapidly, but differences in the type of IFN produced and their magnitude occurs. The expression of IFN-λ is also tissue specific, restricted mainly to epithelial cells (13, 28). Nevertheless, other immune cells also contribute to the expression of IFN-λ in the defense against respiratory viruses.

Epithelial cells represent an important line of defense against respiratory viruses because they are the primary target of viral replication. Experiments with knockout mouse strains that lack receptors for either type I or type III IFN have demonstrated that lung and gastrointestinal tract epithelial cells respond to IFN-λ stimulation, inhibiting viral replication (28).

Nasal epithelial cells are the primary target of many respiratory viruses, and it has been documented that these cells express IFN-λ but not IFN-α/β upon RSV, measles, and mumps viral infection (29), suggesting the preponderance of IFN-λ in virus-infected epithelial cells.

In primary bronchial epithelial cells, the influenza A virus (IAV) was able to induce IFN-λ1 and IFN-λ2/3 before IFN-β (14). Likewise, in normal human bronchial epithelial cells, influenza H1N1 showed a significant increase in IFN-λ in comparison with IFN-α/β (30).

Similar to influenza virus infection, RV infection induced an early expression of IFN-λ1 and IFN-λ2/3 8 h after infection in BEAS-2B and bronchial epithelial cells, whereas IFN-β was detected 24 h after infection (14). Parainfluenza virus 3 (PIV3) has also been reported to induce the expression of IFN-λ1 in BEAS-2 cells 12 h after infection with a peak at 48 h after infection (31).

In the alveolar epithelial cell line A549, RSV induced the expression of IFN-λ1–3, but only human metapneumovirus (HMPV) induced all four isoforms of IFN-λ. In human alveolar type II epithelial cells, IAV induced the expression of IFN-β, IFN-λ2, and IFN-λ1, but IFN-λ1 was induced to the highest degree, suggesting that IFN-λ1 is the main IFN protein induced in alveolar type II epithelial cells in response to IAV (32, 33).

Other respiratory viruses such as hantaviruses are also able to induce IFN-λ in epithelial cells, as indicated by the infection of A549 cells and Vero E6 cell lines with Hantaan virus, Sin Nombre virus, Andes virus, and Prospect Hill virus (34, 35). Furthermore, Hantaan virus is susceptible to the antiviral effects of IFN-λ1 and IFN-λ2 in A549 cells (36) through the activation of the JAK-STAT pathway and ISG expression (37).

DCs also produce IFN-λ in response to respiratory viral infections. It has been shown that HMPV and RSV infections induce higher expression of IFN-λ than type I IFN in human monocyte-derived DCs (38, 39), suggesting a dominant role of IFN-λ in the antiviral response against these viruses. It has been observed that IAV can induce the expression of *ifnlr1* in bone marrow–derived DCs. Moreover, IFN-λ signaling in DCs has been demonstrated to be critical for optimal Ag uptake and processing, upregulation of costimulatory molecules, regulation of IL-10, DC migration from lungs to lymph nodes, and CD8^+^ T cell activation in the context of IAV (16). Plasmacytoid DCs are also responsive and express IFN-λ. This has been demonstrated with the IFN-λ induction occurring after influenza virus H3N2 infection or TLR stimulation (40) and the expression of functional IFN-λ receptors (41).

Alveolar macrophages are a viable source of chemokines and inflammatory and antiviral cytokines in the lung (42, 43). Under basal conditions, they secrete significant levels of chemokines and express functional IFNLR1 (44), which makes them susceptible to activation by IFN-λ-induced antiviral responses. Studies with human monocyte-derived macrophages have shown that influenza infection induces the expression of IFN-λ1. Furthermore, pretreatment of monocyte-derived macrophages with IFN-λ inhibited viral protein expression (44).

### Activation of immune cells by IFN-λ

Neutrophils are important innate immune cells, and they abound in the peripheral blood. Although neutrophils are not reported to produce IFN-λ, they can be activated by the antiviral cytokine. This occurs because mouse and human neutrophils express high levels of the α-subunit (IL-28RA) of the IFN-λ receptor. Thus, treatment with IFN-λ activates the receptor, leading to STAT1 phosphorylation (17). Moreover, the role of IFN-λ in neutrophils not only is limited to the induction of ISG expression but also decreases the capacity of the cells to release reactive oxygen species in response to stimulation with TNF or LPS, suggesting it could prevent inflammation in the respiratory epithelium (45).

Although there is no evidence of a specific function of IFN-λ on B or T cells, B cells and CD8^+^ T cells express the receptor IFNLR1, which has been documented to bind IFN-λ3 and IFN-λ4 (46). Treatment of human B and CD8^+^ T cells with recombinant IFN-λ3 induced the upregulation of ISG expression (15), demonstrating the susceptibility of lymphocytes to activation by IFN-λ. Additional studies on the effects of IFN-λ on immune cells indicate that IFN-λ promotes B cell activation and plasma cell differentiation (47). Overall, further research on the effect of IFN-λ on immune cells is warranted.

## IFN-λ in Experimental Mouse Models

To better understand the physiological relevance of IFN-λ in respiratory viral infections, research using knockout mice lacking a functional IFN-λ receptor (IL-28Rα*^0/0^*) demonstrated that IFN-λ is critical for controlling viral infections such as IAV, influenza B virus, RSV, HMPV, and SARS-CoV-2 (28, 48) and to prevent viral spread from the upper respiratory tract to the lungs (49). Likewise, mice with defective *ifnl2* and *ifnl3* genes had an impaired ability to control infections with IAV (50), stressing the role of IFN-λ as a significant mediator of the antiviral defense in the respiratory tract. Moreover, the administration of exogenous IFN-λ contributes to innate immunity against the influenza virus, activating multiple ISG expression and significantly reducing IAV infection (33, 48, 51) without the increased inflammatory response observed with IFN-α (52, 53). Similarly, mice treated with murine recombinant IFN-λ before HMPV infection showed a significant reduction in lung viral titers, inflammation, and disease. Furthermore, HMPV and RSV are respiratory viruses that induce substantial levels of IFN-λ as early as 12 and 24 h after infection, respectively (54). The use of pegylated IFN-λ has been seen to be effective in combating IAV in mice, given its potent antiviral and immune-modulatory activities without the neutrophilia, lung injury, and lethality occurring as seen with type I IFN (53). However, in vivo data from mice and data from murine airway epithelial cells (AECs) also suggest that treatment with IFN-λ can lead to detrimental effects. For instance, the IFN-λ response elicited by mice infected with IAV may contribute to bacterial superinfection in the lung by inducing the activation of cytokine cell signaling (55) or by decreasing neutrophil infiltration (53). Similarly, in an experimental approach using synthetic RNAs to mimic viral immune activation in mice, the production of IFN-λ by lung DCs resulted in epithelial cell damage, which can increase the susceptibility to secondary bacterial infections (56). Those findings are supported by studies in vitro using murine AECs infected with IAV, where treatment with IFN-λ interfered with AEC growth and differentiation by inducing the protein p53 (57).

Overall, experimental mouse models have contributed to our understanding of the importance and scope of the IFN response. However, more research is necessary to evaluate the time span of IFN-λ biological activity, as well as its beneficial or detrimental effects when used as a broad therapeutic.

## Inhibition of IFN-λ by respiratory viruses

**FIGURE 1.**
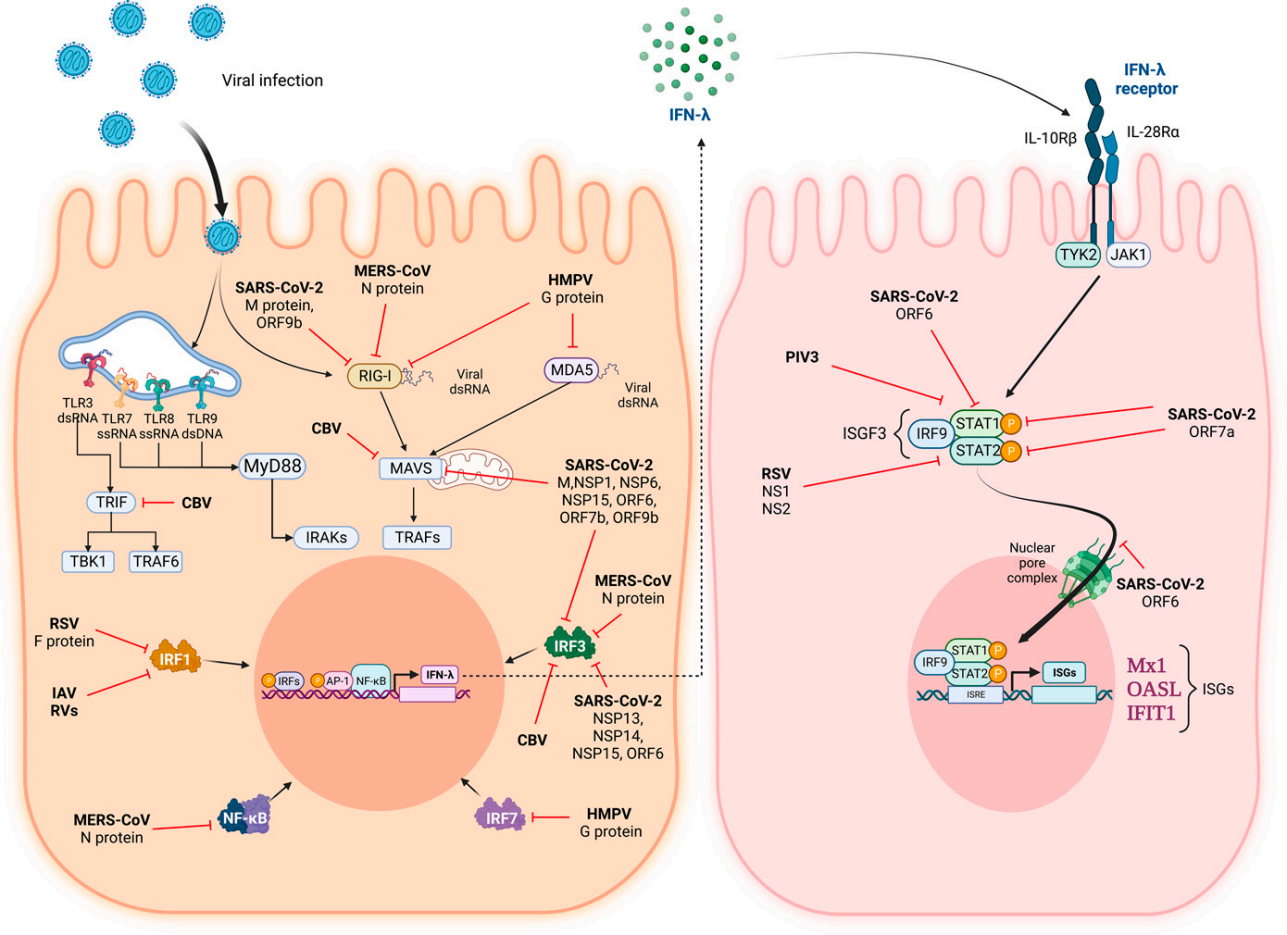
Respiratory viruses have developed mechanisms to inhibit the IFN-λ response. After viral infection, pattern recognition receptors, such as TLRs, RIG-I, and MDA-5, recognize different viral components and trigger pattern recognition receptor–associated signaling pathways that activate NF-κB, AP-1, and IRFs to induce IFN-λ biosynthesis. IFN-λ acts through its dimeric receptor IL10-Rβ and IL-28α. Activation of the IFN-λ receptor induces the activation of the JAK/STAT pathway and the phosphorylation of STAT1 and STAT2, which complex with IRF9 to form the ISG factor 3 (ISGF3) that translocates to the nucleus to induce expression of ISGs to inhibit viral replication. However, respiratory viruses can subvert the IFN-λ response through the expression of specific proteins that target multiple aspects of the induction of the IFN-λ as well as the activation of the IFN-λ antiviral response. Respiratory viruses such as RSV, IAV, MERS-CoV, HMPV, SARS-CoV-2, Coxsackie B virus, and PIV3 possess different structural and nonstructural proteins involved in the blocking of the signaling pathways. F, fusion protein; G, glycoprotein; M, membrane protein; N, nucleocapsid protein; NS1, NS2, NS5, NS6, nonstructural proteins 1, 2, 5, 6; NSP1, NSP6, NSP13, NSP15, nonstructural proteins 1, 6, 13,15; ORF, open reading frame proteins; VP3, viral protein 3. Figure created in BioRender.com.

Respiratory viruses have created diverse mechanisms to evade the IFN response by creating soluble proteins that act as inhibitors of IFN expression or some downstream IFN signaling pathway components (Fig. 1).

Some respiratory viruses, such as IAV and RV, inhibit the expression of IFN-λ through the activation of the epithelial epidermal growth factor receptor by suppressing IRF1 (58). HMPV G protein has been shown to inhibit the expression of IFN-λ1, IFN-λ2/3, and IFN-λ4 in vitro and in vivo by impairing the expression of retinoic acid–inducible gene I (RIG-I), melanoma differentiation–associated protein 5 (MDA-5), MyD88, and IRF7 (54). Similarly, RSV nonstructural proteins 1 and 2 (NS1 and NS2) also antagonize the IFN-λ-mediated antiviral effect by pSTAT2 downregulation (59, 60). Moreover, RSV F protein inhibits IFN-λ by activating epidermal growth factor receptor, suppressing IRF1 (58). PIV3 inhibits IFN-λ downstream signaling in BEAS-2 by decreasing the phosphorylation of STAT1 (31).

Middle East respiratory syndrome coronavirus (MERS-CoV), a potential global threat closely related to SARS-CoV-2, suppresses type I and type III IFN gene expression through its N protein that impedes RIG-I ubiquitination and activation by inhibiting the phosphorylation of IRF3 and NF-κB (61).

SARS-CoV-2 has been shown to use multiple mechanisms to impair innate immunity. For instance, the M protein of this virus interacts with RIG-I, mitochondrial antiviral signaling protein (MAVS), and TBK1, interfering with the formation of multiprotein complexes and blocking the phosphorylation of IRF3 and nuclear translocation, which in turn has an important effect on the expression of type I and type III IFNs (62). Recent evidence also suggests that five SARS-CoV-2 genes (NSP1, NSP6, NSP15, ORF6, and ORF7b) suppress MAVS-induced activation of IFN-λ1 and IFN-λ2/3, contributing to the pathogenicity of the disease (63, 64). Also, the NSP13, NSP14, NSP15, and ORF6 proteins are potent suppressors of the type III IFN response, hampering the nuclear translocation of IRF3, decreasing the chances of reducing viral replication (65–67). ORF6 protein is also an example of the variability of targets that coronaviruses can use to evade the innate immune response. Recent data demonstrated that accessory protein ORF6 localizes at the nuclear pore complex, impeding STAT nuclear translocation, thus inhibiting ISG expression (68). In addition, the ORF9B accessory protein encoded by an alternative open reading frame within the *N* gene has been shown to impede IFN expression by targeting multiple molecules of the axis RIG-I/MDA-5, MAVS, TLR3 TIR-domain-containing adapter-inducing IFN-β (TRIF), and cyclic GMP-AMP synthase-STING (69, 70). Additional mechanisms of IFN-λ activity inhibition by SARS-CoV-2 include usurping of the ubiquitin system to antagonize IFN signaling, by inhibiting nuclear translocation of STAT1 and phosphorylation of STAT2 through ORF7a (71).

Coxsackie B virus, a picornavirus that can replicate in the respiratory tract and cause a variety of ailments, including a sore throat and other respiratory conditions (72), has been shown to inhibit the expression of IFN-λ by impairing the phosphorylation of IRF3 and the expression of TRIF and IPS1 (73). In addition, coxsackie B virus 3 inhibits IFN production by cleavage of eukaryotic initiation factor 4G, MAVS, and TRIF (74).

## IFN-λ as a therapy for respiratory viruses

The initial use of IFN-λ in the treatment of viral infections has revealed advantages over the use of IFN-α2a and IFN-α2b, which have been associated with adverse effects such as fatigue, neurologic signs, autoimmune diseases (75), and proinflammatory effects on immune cells in the respiratory tract (52, 53, 76). In addition, IFN-λ can confer long-lasting protection, whereas IFN-α-mediated protection is short-lived (49), likely because of the stronger and sustained expression of IFN-λ and ISGs (77). However, recent studies indicate that chronic exposure of lung epithelial cells to IFN-λ compromises the lung barrier function, predisposing the host to secondary bacterial infections (78) or impairing lung epithelial regeneration (57). Moreover, in the context of autoimmune disease, IFN-λ has led to immune dysregulation and tissue damage (79). Nevertheless, the benefits possibly outweigh the side effects of using IFN-λ.

Different approaches have been designed to use IFN-λ as an efficacious therapeutic option with a more potent antiviral response than IFN type I. For example, the use of analogs of IFN-λ1 and IFN-λ3 have demonstrated high potency in activating IFN-stimulated response elements (80) as well as the use of recombinant bovine IFN-λ to treat COVID-19 (81). IFN-λ therapy of SARS-CoV-2 infection upregulated ISGs with marginal expression of the ACE2 receptor, positioning IFN-λ as a potential therapeutic agent for COVID-19 (82). Previous studies using primary human bronchial epithelial cells grown in an air–liquid interface that were treated with IFN-λ3 and IFN-λ4 reduced viral replication of human coronaviruses such as human coronavirus 229E and MERS-CoV (11).

Pretreatment of pediatric primary bronchial epithelial cells with high doses of IFN-λ reduced RSV replication (59). Similarly, treatment of nasal epithelial cells with IFN-λ minimized influenza viral loads both in vitro and in vivo (83). Recent work has highlighted the potential of IFN-λ4 as a clinical therapeutic agent after introducing a de novo N-glycosylation that produced an IFN-λ4 variant with robust ISG expression and potency (84), without apoptosis induction. Therefore, further research is warranted using IFN-λ4 to treat viral infections.

### Clinical Trials

In the race for new therapeutic antiviral alternatives, pegylated IFN-λ has been successful in treating viral infections such as hepatitis B virus (85, 86) and other hepatitis viruses (hepatitis C and hepatitis D viruses) under current clinical trials (NCT01741545; NCT05070364). The success of clinical trials against viral hepatitis and the evidence that IFN-λ is one of the main antiviral cytokines released during respiratory infections have led to interest in testing this IFN in the treatment of COVID-19. Currently, there are some active phase 2 and 3 clinical trials to evaluate the effect of PegIFNλ on treating COVID-19. The results of one of the clinical trials (NCT04354259) are encouraging, revealing that in outpatients with COVID-19 treated by s.c. injection of pegylated IFN-λ at a dosage of 180 µg within 7 d of symptom onset or the first positive swab, viral decline was accelerated and the number of patients with viral clearance by day 7 was increased (87). This clinical trial represents the first of its type to evaluate the effect of IFN-λ in the respiratory tract, opening the door to finding new effective therapeutic alternatives against COVID-19 or prophylactic treatments, as indicated in the clinical trial (NCT04344600). The promising results of IFN-λ against SARS-CoV-2 demonstrate the potential for use of this IFN to treat other respiratory viral infections for which no vaccines are available. The ongoing clinical trials of IFN-λ (NCT04534673, NCT04727424, and NCT04967430) for COVID-19 should contribute significantly to the landscape of new effective and safe therapeutic alternatives. Due to the low inflammatory profile and the potent response in inducing ISGs, the pegylated IFN-λ may be a promising candidate to treat different respiratory viral infections.

## Conclusions

This review highlights the relevance of IFN-λ in respiratory viral infections, indicating that although viruses induce the expression of this cytokine in the respiratory tract, they have also developed mechanisms to evade its response. Nevertheless, more research is needed to unveil the role of IFN-λ in other immune cells and to determine how they contribute to the innate and adaptive responses during a respiratory viral infection. Moreover, research is warranted to understand the mechanisms underlying the detrimental effects of IFN-λ, leading to opportunistic bacterial infections and disease severity. Taking those effects into consideration, the use of IFN-λ as a therapeutic approach still could represent an opportunity in the treatment and prevention of respiratory viral infections. Although most clinical trials have focused on the use of IFN-λ in treatments for chronic hepatitis, IFN-λ has emerged as a potential target to treat and prevent disease caused by SARS-CoV-2, demonstrating the potential likelihood of its use in other respiratory viral infections. The lack of IFN-λ clinical trials for respiratory viruses other than SARS-CoV-2 indicates that there are multiple opportunities for exploration on the horizon. This is relevant because new respiratory viral strains evolve each year, resulting in pandemics or seasonal infections that can severely affect immunocompromised patients as well as healthy individuals, which may cause secondary infections or even death.
